# The Genus *Chlorosplenium* (Helotiales, Leotiomycetes) from China with Notes on *C. chlora* Complex

**DOI:** 10.3390/life11111167

**Published:** 2021-11-01

**Authors:** Huan-Di Zheng, Wen-Ying Zhuang

**Affiliations:** State Key Laboratory of Mycology, Institute of Microbiology, Chinese Academy of Sciences, Beijing 100101, China; zhenghd@im.ac.cn

**Keywords:** Chlorospleniaceae, DNA barcode, fungi, novel taxa, species diversity, saprotrophic

## Abstract

The small fruitbodies of *Chlorosplenium* are greenish yellow and mainly grow on woody substrates. The species diversity of the genus in China was investigated based on specimens formerly deposited in the Herbarium Mycologicum Academiae Sinicae as well as new collections gained in recent years. Our phylogenetic results revealed the species diversity of the genus is underestimated and the commonly known *Chlorosplenium chlora* is a species complex. Based on morphology studies and sequence analyses of three regions (ITS, LSU and *RPB1*), the Chinese collections represent two new species which are described and illustrated here as *C. sinicum* and *C. sinochlora*. *Chlorosplenium fusisporum* is quite possibly a species of the genus *Chlorociboria*, and *C. hyperici-maculati* should be excluded from the genus.

## 1. Introduction

*Chlorosplenium* Fr. is an inoperculate discomycete genus in Helotiales (Leotiomycetes, Ascomycota, Fungi). It was established in 1849, typified by *C. chlora* (Schwein.) M.A. Curtis, and originally included three species (*Peziza chlora* Schwein., *P. chlorascens* Schwein. and *P. torta* Schwein.) [1]. Although only three species were firstly involved, their taxonomic and nomenclatural changes were complicated which were detailed by Dixon in his monographic treatments of the genera *Chlorsplenium*, *Chlorociboria* Seaver and *Chlorencoelia* Dixon [1,2]. In this study, Dixon’s taxonomic concept of *Chlorosplenium* is generally followed.

Many saprotrophic inoperculate cup-fungi have been placed in *Chlorosplenium* on the basis of the phenotypic character grayish- to greenish-yellow apothecia, and 57 names were published under the genus (http://www.indexfungorum.org/Names/Names.asp (accessed on 15 August 2021)). However, many of them are obviously not congeneric with the type species *C. chlora*, and they were even scattered in 11 different families according to the recent taxonomic treatments [3]. *Chlorosplenium* was once treated as a member of Helotiaceae or Dermateaceae [1,4]. According to the results of multi-locus phylogenetic analysis, the genus appeared as a clade distinct from any other fungal groups, thus a new family Chlorospleniaceae was proposed [5].

Five species are currently known in *Chlorosplenium* [4], i.e., *C. chlora*, *C. hypochlorum* (Berk. & M.A. Curtis ex W. Phillips) J.R. Dixon [1,2], *C. cenangium* (De Not.) Korf [6], *C. fusisporum* Liou & Z.C. Chen [7], and *C. hyperici-maculati* Svrček [8], among which two were previously recorded from China [9]. Species of the genus are somewhat easily recognized in the field, and are characterized by discoid to shallowly cupulate, grayish to greenish yellow apothecia with enrolled margin, usually sessile, reaching a size of 0.5–4 mm in diameter; hyphal extensions of different lengths present on receptacle surface or having a glabrous surface; ectal excipulum of textura angularis with the outer cell layer brown and the inner cells subhyaline to hyaline, medullary excipulum of textura intricata with hyaline hyphae and non-gelatinous tissues, subhymenium usually present and appearing as a densely interwoven hyphal layer; asci cylindric-clavate and containing eight ascospores; ascospores subcylindrical, hyaline and commonly unicellular; and paraphyses filiform and not or slightly exceeding the asci. Members of the genus are wood-inhabiting.

During our work on species diversity of Dermateaceae and its allies in China, herbarium specimens and recent collections of *Chlorosplenium* were studied based on morphological features and DNA sequence data. As a result, the occurrence of *C. chlora* in China should be re-considered, and two new species are discovered. The taxonomic position of the previously recorded *C. fusisporum* Liou & Z.C. Chen is doubtful based on the original description and illustration [7] and is tentatively excluded from the genus.

## 2. Materials and Methods

Specimens examined were all deposited in the Herbarium Mycologicum Academiae Sinicae (HMAS) and collected during 1933−2020 from Anhui, Chongqing, Guangdong, Guangxi, Henan, Hubei, Hunan, Jiangxi, Shaanxi, and Yunnan provinces of China. Photographs of fresh apothecia were taken by a Canon PowerShot G16 digital camera. Dried apothecia were rehydrated with distilled water and sectioned at a thickness of 15−20 μm with a Yidi YD-1508A freezing microtome (Jinhua, China). Mounting media was lactophenol cotton blue. Iodine reactions of ascus apical apparatus were tested in Melzer’s reagent and Lugol’s solution with or without 3% KOH solution pretreatment. An Olympus BH-2 microscope (Tokyo, Japan) was used for microscopic examination and measurements. Microscopic photographs were taken using a ZEISS Axiocam 305 color microscope camera (Göttingen, Germany) attached to a Zeiss Axioskop 2 Plus microscope (Göttingen, Germany).

Genomic DNA was extracted from dried apothecia using Plant Genomic DNA Kit (TIANGEN Biotech. Co., Beijing, China). Primer pairs used for amplification and sequencing included ITS1&ITS4 or ITS5&ITS4 [10] for the nuclear internal transcribed spacers (ITS), LR0R [11] and LR5 [12] for D1/D2 domain of the nuclear large subunit ribosomal DNA (LSU), RPB1-Af [13] and RPB1-Cr [14] for RNA polymerase II largest subunit gene (*RPB1*). PCR products were purified and sequenced at Beijing Tianyi Huiyuan Bioscience and Technology, China.

Newly generated sequences were assembled using SeqMan (DNASTAR, Lasergene 7.1.0) and BioEdit 7.0.5.3 [15]. The new sequences were deposited at GenBank and additional sequences used for phylogenetic analyses were downloaded from GenBank (Table 1). The sequence matrix was aligned by MUSCLE [16] and manually edited using BioEdit 7.0.5.3 [15]. Introns and ambiguous sites were removed and excluded from the analysis process. *Dermea acerina* and *Pezicula carpinea* were chosen as outgroup taxa.

Maximum likelihood (ML) and Bayesian inference (BI) analyses were conducted with RAxML 8.0 [17] and MrBayes 3.1.2 [18], respectively. Parameters of the ML analysis were set as default, and statistical support values were obtained from 1000 replicates of bootstrap (BP) tests. The BI analysis was performed using the best-fit evolutionary model estimated by MrModeltest 2.3 [19] with the Akaike information criterion. Two parallel runs of four simultaneous chains of Markov Chain Monte Carlo were performed for 2,000,000 generations with the trees sampled every 100 generations. The first quarter of sampled trees were discarded as the burn-in phase, and the remaining trees were used for calculating posterior probabilities (PP) in the majority rule consensus tree. The phylogenetic trees were visualized in FigTree 1.4.4 [20].

## 3. Results

### 3.1. Phylogenetic Analyses

**Figure 1:**
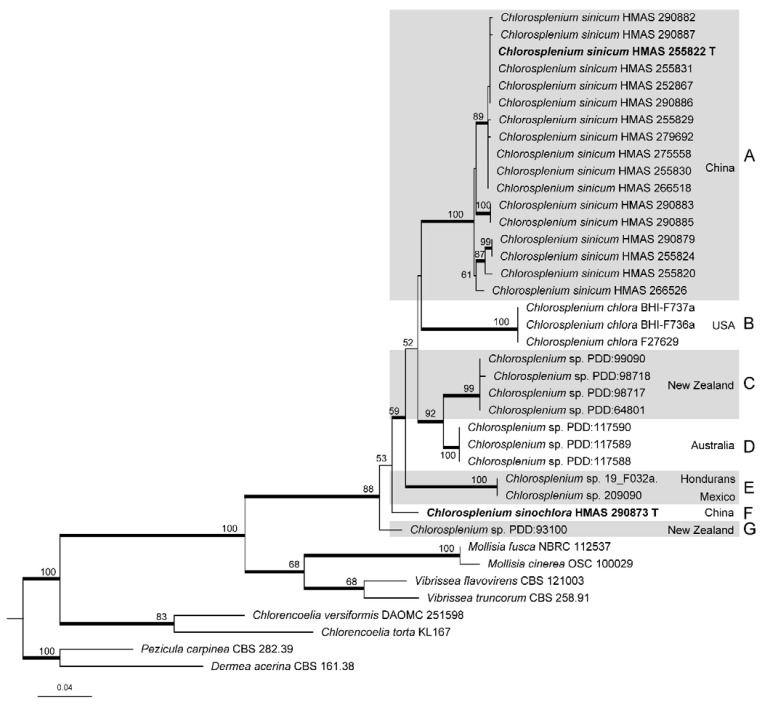
Maximum likelihood phylogenetic tree inferred from ITS sequences including *Chlorosplenium* collections and related taxa. Bootstrap support values ≥ 50% are shown at nodes. Posterior probability values ≥ 0.90 of BI analysis are indicated as thick branches. Holotypes are shown in bold and marked with the letter “T”.

The final ITS matrix included 31 sequences of *Chlorosplenium* and eight of related taxa, and the alignment contained 595 nucleotide sites. GTR+I+G was selected as the best-fit model for BI analysis. The phylogenetic trees yielded by ML and BI analyses are similar in topology, and the ML tree is presented in Figure 1 with bootstrap values at nodes. All the *Chlorosplenium* sequences clustered together (MLBP/BIPP = 88%/1.00) that confirmed the monophyly of the genus. Seven well-supported clades/lineages (A–G) were recognized, including the two proposed new taxa from China.

Phylogenetically, all collections of *C. sinicum* were strongly supported in clade A which were further divided into a few subclades. *Chlorosplenium sinochlora* appeared as an isolated lineage F not closely related to the others. *Chlorosplenium chlora* from the USA materials clustered in clade B, and they were typical representatives of the species. Clades C, D, E and G consisted of unidentified samples of *Chlorosplenium* from New Zealand, Australia, Mexico, and Honduras, which were shown as *Chlorosplenium* spp. We suspect they might represent either un-named species or the existing species which have not been connected with any DNA sequences.

**Figure 2:**
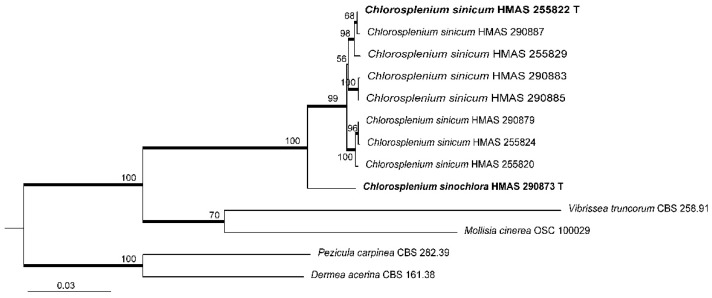
Maximum likelihood phylogenetic tree inferred from combined ITS-LSU-*RPB1* sequences of selected *Chlorosplenium* collections and related taxa. Bootstrap support values ≥ 50% are shown at nodes. Posterior probability values ≥ 0.90 of BI analysis are indicated as thick branches. Holotypes are shown in bold and marked with the letter “T”.

Due to the lack of LSU and *RPB1* sequence data in most *Chlorosplenium* collections, the combined ITS-LSU-*RPB1* dataset only included 13 sequences of the Chinese material, as well as four related inoperculate discomycete taxa. The final alignment comprised 2234 sites including gaps, of which 655 bp for ITS, 825 bp for LSU and 754 bp for *RPB1*. The best-fitting model for BI analysis was GTR+I for the multi-locus sequence data. Figure 2 showed the ML tree resulted from ITS-LSU-*RPB1* sequence analysis with bootstrap values higher than 50%. Similar to the ITS analysis, *C. sinicum* is divided into several subgroups.

### 3.2. Taxonomy

#### 3.2.1. *Chlorosplenium sinicum* H.D. Zheng & W.Y. Zhuang, sp. nov.

- Fungal Names—FN570862
- Etymology—The specific epithet refers to the type locality of the species.
- Holotype—CHINA, Hunan, Yizhang, Mang Mt., on rotten wood, 28 Oct. 2015, Z.Q. Zeng et al. 10345 (HMAS 255822).

**Figure 3:**
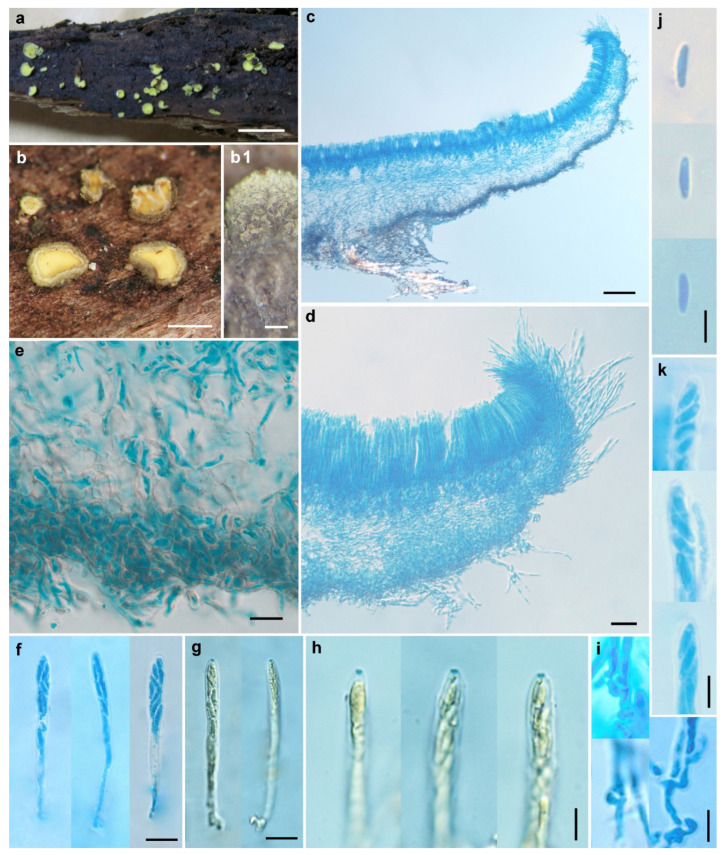
*Chlorosplenium sinicum* H.D. Zheng & W.Y. Zhuang (HMAS 255822, holotype). (**a**) Fresh apothecia on natural substrate; (**b**) Dry apothecia; (**b1**) Receptacle surface of dry apothecium, showing groups of hairs; (**c**,**d**) Longitudinal section of apothecium; (**e**) Excipular structure at flank; (**f**,**g**) Asci; (**h**) IKI reaction of ascus apical rings; (**i**) Croziers at ascus base; (**j**) Ascospores; (**k**) Ascospores in asci. Scale bars: (**a**) = 5mm; (**b**) = 1 mm; (**b1**) = 0.2 mm; (**c**) = 100 μm; (**d**) = 20 μm; (**e**–**g**) = 10 μm; (**h**–**k**) = 5 μm. Mounting media: (**c**–**f**,**i**–**k**) lactophenol cotton blue; (**g**,**h**) Melzer’s reagent.

Description—Apothecia scattered to gregarious, sessile, cupulate to discoid, margin slightly incurved when fresh, 0.5–4 mm in diameter; hymenium surface is greenish yellow, green tint becoming faded after drying; receptacle surface concolorous or darker, covered by hairs, whitish at margin, becoming greyish brown toward the base. Hairs on margin and upper flanks, cylindrical, round at the apex, multi-septate, smooth, straight to slightly flexuous, thin- to slightly thick-walled, hyaline at margin and light brown to brown at flanks, 2–4 μm wide, up to 90 μm long at margin and 8–45 μm long at flank, long hairs growing in groups, short hairs interspersed among those groups. Anchoring hyphae brown, thick-walled, 2.5–3.5 μm wide, walls 0.8–1 μm thick, slightly refractive, forming a layer of textura intricata at the base of apothecia, 40–140 μm thick. Ectal excipulum of two layers, less differentiated at margin; outer layer of textura angularis, 14–25 μm thick, cells slightly thick-walled, light brown to brown, nearly isodiametric, 3.5–8 μm in diam., walls 0.5–1 μm thick; inner layer of textura angularis and of textura prismatica at margin, 17–40 μm thick, cells thin-walled, subhyaline, 7–12 × 5–12 μm. Medullary excipulum of textura intricata, 20–220 μm thick, hyphae hyaline, thin- to slightly thick-walled, 2–4 μm wide. Subhymenium 15–30 μm thick. Hymenium 60–75 μm thick. Asci arising from croziers, 8-spored, uniseriate to irregularly biseriate, cylindric-clavate, J+ in Melzer’s reagent and Lugol’s solution without KOH pretreatment, visible as two blue lines, 45–66 × 4–6 μm. Ascospores subcylindrical, usually asymmetrical, aseptate, hyaline, smooth, with two or more minute guttules, 5.5–7.7 × 1.5–2.2 μm. Paraphyses filiform, slightly enlarged at apex, hyaline, 2–3 μm broad at apex and 1.5–2 μm below, slightly exceeding the asci by 5–10 μm (Figure 3).

Other specimens examined—CHINA, Anhui, Huang Mt., on rotten wood, 27 August 1957, S.C. Teng 5121 (HMAS 20093); *ibid.*, 30 August 1957, S.C. Teng 5239 (HMAS 20500); Anhui Jiuhua Mt., on hard wood, 18 September 1933, Anhui, S.C. Teng 495 (HMAS 9326); Anhui, Jinzhai, Tiantangzhai, alt. 900−1000 m, on rotten wood, 23 August 2011, S.L. Chen et al. 7805 (HMAS 266626); Anhui, Jinzhai, Tiantangzhai, alt. 900−1000 m, on wet hard wood, 24 August 2011, S.L. Chen et al. 7879 (HMAS 252866); Chongqing, Jinyun Mt., alt. 823 m, on rotten or hard wood, 22 October 2020, H.D. Zheng et al. 12556, 12557, 12558, 12559, 12560, 12561 (HMAS 290886, 255828, 255829, 255830, 290887, 255831); Fujian, on rotten wood, S.C. Teng 1977 (HMAS 56523); Guangdong, Fengkai, alt. 300 m, on hard wood, 27 October 1998, W.Y. Zhuang & Z.H. Yu 2854 (HMAS 266519); *ibid.*, 28 October 1998, W.Y. Zhuang & Z.H. Yu 2861 (HMAS 82038); Guangdong, Huidong, Guohua Forest Farm, alt. 800−900 m, on rotten wood, 17 October 1998, W.Y. Zhuang & Z.H. Yu 2764 (HMAS 252350); Guangdong Maoming, Dawuling, alt. 1400 m, on rotten wood, 22 October 1998, W.Y. Zhuang & Z.H. Yu 2810 (HMAS 266520); Guangdong, Shaoguan, Chebaling, on rotten wood, 31 October 2015, Z.Q. Zeng et al. 10482 (HMAS 255826); Guangdong, Shixing, Chebaling, Xianrendong, on rotten bark, 1 November 2015, Z.Q. Zeng et al. 10543 (HMAS 290882); Guangdong, Zhaoqing, Dinghu Mt., alt. 150 m, on rotten wood, 10 October 1998, W.Y. Zhuang & Z.H. Yu 2678 (HMAS 266521); Guangdong, Zhaoqing, Dinghu Mt., on rotten wood, 8 September 1958, J.H. Yu et al. 66 (HMAS 28256); Guangxi, Wuming, Daming Mt., alt. 1200 m, on rotten wood, 18 December 1997, W.Y. Zhuang & X.Q. Zhang 1796 (HMAS 74881); Guangxi, Wuming, Daming Mt., alt. 1100 m, on rotten wood, 19 December 1997, W.Y. Zhuang & X.Q. Zhang 1852 (HMAS 74882); Guangxi, Wuming, Daming Mt., alt. 900 m, 20 December 1997, W.Y. Zhuang 1863 (HMAS 266616); Henan, Jigong Mt., alt. 400 m, on hard wood, 13 November 2003, W.Y. Zhuang & Z.H. Yu 5080 (HMAS 266517); *ibid.*, 14 November 2003, W.Y. Zhuang & Z.H. Yu 5089 (HMAS 266518); Hubei, Shennongjia, on rotten wood, 17 August 1984, H.Z. Li (HMAS 56467); *ibid.*, 19 September 2014, H.D. Zheng et al. 9822 (HMAS 275558); Hunan, Yizhang, Mang Mt., Jiangjunzhai, on rotten wood, 26 October 2015, Z.Q. Zeng et al. 10249 (HMAS 255820); Hunan, Yizhang, Mang Mt., Jiangjunzhai, on rotten wood, 27 October 2015, Z.Q. Zeng et al. 10300 (HMAS 255821); Hunan, Yizhang, Mang Mt., on rotten wood, 28 October 2015, Z.Q. Zeng et al. 10355 (HMAS 255823); Hunan, Yizhang, Mang Mt., on rotten wood, 29 October 2015, Z.Q. Zeng et al. 10398, 10416, 10417, 10433, 10444 (HMAS 255824, 255825, 290878, 290879, 290881); Jiangxi, Lushan Botanic Garden, on wood, 19 October 1996, W.Y. Zhuang & Z. Wang 1465, 1466 (HMAS 252352, 266522); Jiangxi, Sanqing Mt., alt. 700 m, 19 September 2006, W.Y. Zhuang & J. Luo 6873 (HMAS 266523); Shaanxi, Qinling, Xiangxiyuan, on rotten wood, September 2015, P.J. Han, 8260 (HMAS 279692); Yunnan, Chuxiong, Zixi Mt., alt. 2120 m, on rotten wood, 8 Aug. 2016, X.H. Wang et al. YN16-34 (HMAS 290883); Yunnan, Hekou, Dawei Mt., alt. 1900 m, on rotten wood, 5 November 1999, W.Y. Zhuang & Z.H. Yu 3312 (HMAS 266524); Yunnan, Gaoligong Mt., Baihualing, on hard wood, 15 September 2017, H.D. Zheng et al. 11373, 11395 (HMAS 290884, 255827); Yunnan, Gaoligong Mt., Baihualing, Jinchanghe, on rotten wood, 17 September 2017, H.D. Zheng et al. 11441 (HMAS 290885); Yunnan, Menghai, Mangao, alt. 1300 m, on rotten wood, 22 October 1999, W.Y. Zhuang & Z.H. Yu 3199, 3252 (HMAS 252351, 266525); Yunnan, Pingbian, Dawei Mt., alt. 1900 m, 4 November 1999, W.Y. Zhuang & Z.H. Yu 3259 (HMAS 266526); Yunnan, Simao, 6 May 1957, L.W. Xu & G.Z. Wang 989 (HMAS 24109); Yunnan Simao, Caiyanghe, alt. 1300 m, on rotten wood, 13 October 1999, W.Y. Zhuang & Z.H. Yu 1863 (HMAS 266617).

Notes—*Chlorosplenium sinicum* is similar to *C. chlora* in gross morphology and microscopic characters, but it is distinct in receptacle surface fibrillose with conspicuous hairs and the DNA sequence data. According to Dixon (1974), the receptacle surface of *C. chlora* is glabrous [1]. In the phylogenetic tree (Figure 1), the Chinese collections of *C. sinicum* formed a well-supported clade independent from other species. The samples of the fungus further grouped into a few subclades (Figure 1 and Figure 2) and sequence variations within species were ranged 18 bp including 10 gaps for ITS, 0–3 bp for LSU, and 0–6 bp for *RPB1*. Morphological distinctions were not detected among the collections. We treat the above divergences as intra-specific variations.

#### 3.2.2. *Chlorosplenium* *sinochlora* H.D. Zheng & W.Y. Zhuang, sp. nov.

- Fungal Names—FN570863
- Etymology—The specific epithet refers to the origin of the species and its similarity with *C. chlora*.
- Holotype—CHINA, Hunan, Yizhang, Mang Mt., Jiangjunzhai, on rotten bark, 27 October 2015, Z.Q. Zeng et al. 10286 (HMAS 290873).

**Figure 4:**
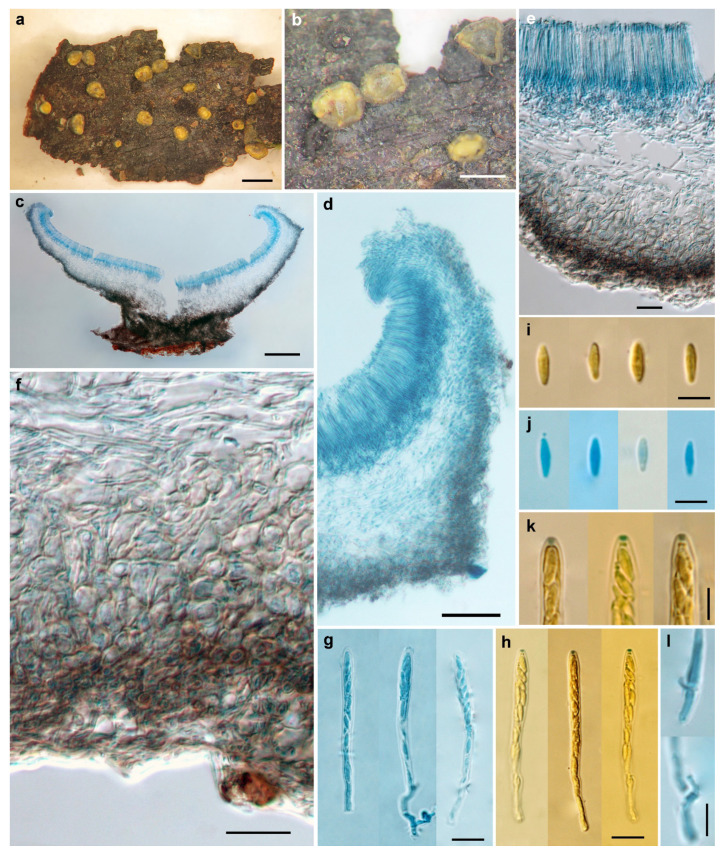
*Chlorosplenium sinochlora* H.D. Zheng & W.Y. Zhuang (HMAS 290873, holotype). (**a**,**b**) Dry apothecia on natural substrate; (**c**) Longitudinal section of apothecium; (**d**) Structure of margin and upper flanks; (**e**) Structure of lower flanks; (**f**) Detailed excipular structure at flank; (**g**,**h**) Asci; (**i**,**j**) Ascospores; (**k**) IKI reaction of ascus apical rings; (**l**) Croziers at ascus base. Scale bars: (**a**) = 2 mm; (**b**) = 1 mm; (**c**) = 200 μm; (**d**) = 50 μm; (**e**,**f**) = 20 μm; (**g**,**h**) = 10 μm; (**i**–**l**) = 5 μm. Mounting media: (**c**–**g**,**j**,**l**) lactophenol cotton blue; (**h**,**i**,**k**) Melzer’s reagent.

Description—Apothecia scattered, subsessile to sessile, shallow-cupulate to discoid, margin slightly incurved when dry, 0.5–2 mm in diam; the hymenium surface is yellowish green, with the green tint becoming obscure when dry; the receptacle surface is greyish green, and greyish black when dry, nearly glabrous. Ectal excipulum of two layers, less differentiated at margin, 20–95 μm thick; cortical layer with short sparsely hyphal protrusions, scattered on the surface of the upper flanks, becoming longer and forming a covering layer from the middle flanks to the base, 5.5–14 μm thick, forming a few minute pustules, hyphae thick-walled, 2.5–3 μm wide, walls refractive, 0.7–1 μm thick; outer layer of textura globulosa-angularis, 20–70 μm thick, cells thick-walled, brown, nearly isodiametric, 3–8 μm in diam., walls 0.5–1.5 μm thick; inner layer of textura angularis, 20–40 μm thick, cells thick-walled, subhyaline to light brown, 5.5–14 × 5.5–11 μm, walls 0.5–1.5 μm thick. Medullary excipulum of textura intricata, loosely arranged, 15–125 μm thick, hyphae hyaline, thin-walled, 2–4 μm wide. Subhymenium 15–25 μm thick. Hymenium 60–70 μm thick. Asci arising from croziers, 8-spored, uniseriate or irregularly biseriate, cylindric-clavate, J+ in Melzer’s reagent and Lugol’s solution without KOH pretreatment, visible as two blue lines, 45–53 × 3.5–4.5 μm. Ascospores subcylindrical, aseptate, hyaline, smooth, with two or more minute guttules, 5–6.5 × 1.8–2.2 μm. Paraphyses filiform, slightly enlarged at apex, hyaline, 2.5–3 μm broad at apex and 1.5–2 μm below, slightly exceeding the asci by 3–8 μm (Figure 4).

Other specimens examined—CHINA, Hunan, Yizhang, Mang Mt., Jiangjunzhai, on rotten wood, 27 October 2015, Z.Q. Zeng et al. 10302, 10311 (HMAS 290875, 290876); *ibid.*, on rotten bark, 10316 (HMAS 290877); Hunan, Yizhang, Mang Mt., on rotten bark, 29 October 2015, Z.Q. Zeng et al. 10413, 10442 (HMAS 290874, 290880).

Notes—In the ITS sequence analysis, *C. sinochlora* appeared as a separate lineage in the phylogenetic tree (Figure 1). Morphologically, it is similar to *C. chlora* but differs in solitary apothecia, narrower asci (45–53 × 3.5–4.5 μm vs. 45–55 × 5–6 μm), overlapped but slightly shorter and wider ascospores (5–6.5 × 1.8–2.2 μm vs. 6–7 × 1.5–2 μm) [1], as well as the ITS sequence variations. It is also similar to *C. sinicum* in ascus and ascospore morphology, but the latter differs in the hairy receptacle surface, lighter cells in outer ectal excipulum, and distinct ITS, LSU and *RPB1* sequences.

#### 3.2.3. Excluded and Doubtful Species of *Chlorosplenium*

##### *Chlorosplenium fusisporum* Liou & Z.C. Chen

**Figure 5:**
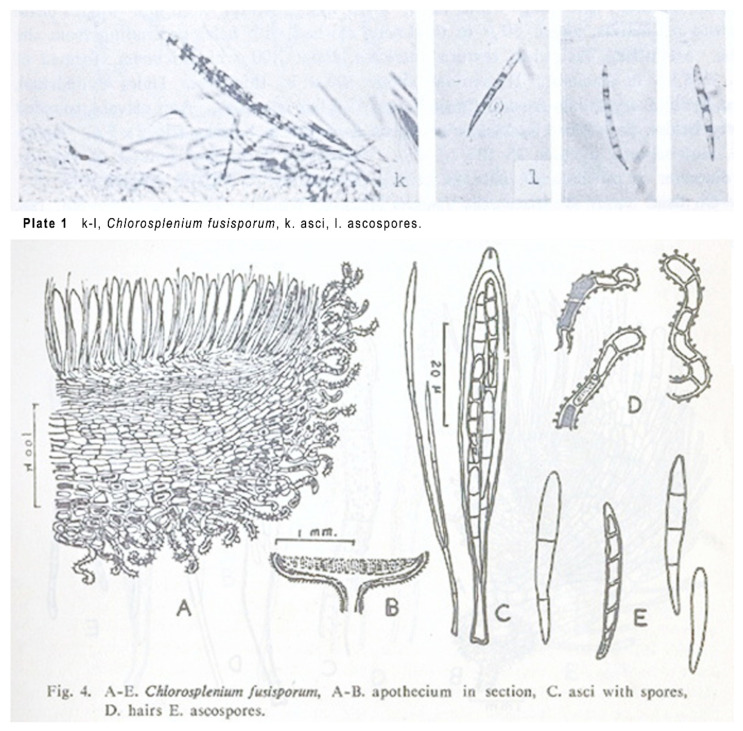
Original illustrations of *Chlorosplenium fusisporum* (From Liou and Chen 1977 [7]).

Notes—*Chlorosplenium fusisporum* was originally described from Taiwan, China [7]. It is characterized by green apothecia and with a centrally placed stipe, ectal excipulum with coiling or undulate hairs encrusted by green granules, acerose ascospores with 1–3 septa, 20–35 × 2.5–3.5 μm (Figure 5). Although we did not access the voucher specimen of *C.* *fusisporum* which lacks sequence data, the combination of apothecial color, ectal excipulum structure and short and granulate hairs on the receptacle surface strongly suggests its affinity to *Chlorociboria*. The type material of the fungus has not been examined, and therefore formal nomenclatural treatment is not proposed for the time being.

##### *Chlorosplenium hyperici-maculati* Svrček

Notes—*Chlorosplenium hyperici-maculati* was originally described from Slovakia on stem of *Hypericum maculatum* [8]. In his original description, Svrček indicated that “the beautiful color of this discomycete is conspicuously deep green in all outer parts of apothecia, and the disc has an almost velvet appearance.” As to its similar or closely related fungus, he compared *C.* *hyperici-maculati* with *C. aeruginellum* (P. Karst.) P. Karst., which is now being placed in the genus *Dasyscyphus* Nees ex Gray [≡ *Dasyscyphus aeruginellus* (P. Karst.) Korf & J.R. Dixon] belonging to a different family [1]. Judged by the original description and illustration of the fungus, we conclude that *C. hyperici-maculati* should be excluded from *Chlorosplenium* in a modern sense.

#### 3.2.4. Key to Accepted Species of *Chlorosplenium*

1. Asci longer than 70 μm long ................................................................................................... 2

1. Asci shorter than 70 μm long .................................................................................................. 3

 2. Ascospores 12−14 × 4−5 μm ............................................................................ *C. cenangium*

 2. Ascospores (8−)9−14(−15) × 2−4 μm ........................................................... *C. hypochlorum*

3. Receptacle surface fibrillose, hairs clearly visible at least in margin and upper flank,

 ascospores 5.5–7.7 × 1.5–2.2 μm ............................................................................ *C. sinicum*

3. Receptacle surface nearly glabrous ........................................................................................ 4

 4. Apothecia solitary to gregarious, asci (40−)45−55(−60) × (4−)5−6(−7) μm, ascospores

 (5−)6−7(−9) × 1.2−2(−3) μm .................................................................................... *C. chlora*

 4. Apothecia solitary, asci 45–53 × 3.5–4.5 μm, ascospores 5–6.5 × 1.8–2.2 μm ....................

 .............................................................................................................................. *C. sinochlora*

## 4. Discussion

*Chlorosplenium* is a small genus in Helotiales and contains five species. Due to the small size of the apothecia and inconspicuous appearance, members of the genus were not reported frequently. Among the accepted species of the genus, the type species, *C.* *chlora*, was the most widely reported. It was originally described from USA and reported also from Canada, China, Costa Rica, India, Indonesia, Jamaica, Japan, Korea, and Russia [1,9,21,22,23]. *Chlorosplenium* *cenangium* was initially described from Italy [24] and later recorded from Spain [25]. *Chlorosplenium* *hypochlorum* is known from its type locality in Cuba as well as Jamaica and Mexico [1]. *Chlorosplenium* *sinicum* and *C. sinochlora* are reported only from China in the present study. Furthermore, a few ITS sequences of un-named species of the genus deposited at GenBank were derived from Australia and New Zealand.

Morphological details provided in the literature are not enough for accurate species identification of *Chlorosplenium* because macro- and microscopic features are usually overlapping. Molecular study was seldom carried out in the genus and it is necessary to use molecular approaches to facilitate species recognition. Concerning the taxonomic characters, structure of excipulum and morphology of asci and ascospores are useful in species identification. According to the results of our phylogenetic analyses, inter-specific variations of ITS rDNA, the universal barcode for fungi, are higher than those of the protein-coding gene *RPB1* in *Chlorosplenium*, which is suitable for species distinction. Based on the results of the detailed morphological comparison and phylogenetic analyses, the Chinese collections previously identified as *C. chlora* were proved to represent two novel species. Thus, *C. chlora* should be excluded from the fungal species catalogue of China [9]. Its occurrences in other Asian countries are also doubtful.

Based only upon specimens collected from China, this study has shown that the worldwide species diversity of *Chlorosplenium* has not been investigated using modern methods, and many hidden species are expected to be discovered. Future studies based on large-scale samplings combining with ecological, morphological, and molecular data will lead to a better understanding of the species diversity and phylogeny of the genus.

## Figures and Tables

**Table 1:** Sequences of *Chlorosplenium* and related taxa used in this study.

Name	Voucher/Isolate	Country	GenBank Number		
			ITS	LSU	*RPB1*
*Chlorosplenium chlora* (Schwein.) M.A. Curtis	BHI-F736	USA	MG553993		
*C. chlora*	BHI-F737	USA	MG553994		
*C. chlora*	F27629	USA	MZ312166		
*C. sinicum* H.D. Zheng & W.Y. Zhuang	HMAS 279692 (8260)	China	MK425600		
*C. sinicum*	HMAS 266518	China	MK425599		
*C. sinicum*	HMAS 252867	China	**MZ914636**		
*C. sinicum*	HMAS 266526	China	**MZ914637**		
*C. sinicum*	HMAS 275558 (9822)	China	**MZ914635**		
*C. sinicum*	HMAS 290882 (10543)	China	**MZ914627**		
*C. sinicum*	HMAS 290886 (12556)	China	**MZ914630**		
*C. sinicum*	HMAS 255830 (12559)	China	**MZ914632**		
*C. sinicum*	HMAS 255831 (12561)	China	**MZ914634**		
*C. sinicum*	HMAS 255822 **^T^** (10345)	China	**MZ914624**	**MZ920115**	**MZ945729**
*C. sinicum*	HMAS 255829 (12558)	China	**MZ914631**	**MZ920119**	**MZ945733**
*C. sinicum*	HMAS 290887 (12560)	China	**MZ914633**	**MZ920120**	**MZ945734**
*C. sinicum*	HMAS 255820 (10249)	China	**MZ914623**	**MZ920114**	**MZ945728**
*C. sinicum*	HMAS 255824 (10398)	China	**MZ914625**	**MZ920116**	**MZ945730**
*C. sinicum*	HMAS 290879 (10433)	China	**MZ914626**	**MZ920117**	**MZ945731**
*C. sinicum*	HMAS 290885 (11441)	China	**MZ914628**	**MZ920118**	**MZ945732**
*C. sinicum*	HMAS 290883 (YN16-34)	China	**MZ914629**	**MZ920121**	**MZ945735**
*C. sinochlora* H.D. Zheng & W.Y. Zhuang	HMAS 290873 **^T^** (10286)	China	**MZ914622**	**MZ920122**	**MZ945736**
*Chlorosplenium* sp.	HONDURAS19-F032	Honduras	MT571528		
*Chlorosplenium* sp.	209090	Mexico	MG976227		
*Chlorosplenium* sp.	PDD:117588	Australia	MW191764		
*Chlorosplenium* sp.	PDD:117589	Australia	MW191759		
*Chlorosplenium* sp.	PDD:117590	Australia	MW191760		
*Chlorosplenium* sp.	PDD:64801	New Zealand	MW191757		
*Chlorosplenium* sp.	PDD:98717	New Zealand	MW191758		
*Chlorosplenium* sp.	PDD:98718	New Zealand	MW191763		
*Chlorosplenium* sp.	PDD:99090	New Zealand	MW191761		
*Chlorosplenium* sp.	PDD:93100	New Zealand	MW191762		
*Chlorencoelia torta* (Schwein.) J.R. Dixon	KL167	China	LT158424		
*C*. *versiformis* (Pers.) J.R. Dixon	DAOMC 251598	Canada	MH457140		
*Dermea acerina* (Peck) Rehm	CBS 161.38/AFTOL-ID 941	Canada	MH855942	MH867440	DQ471164
*Mollisia cinerea* (Batsch) P. Karst.	OSC 100029/AFTOL-ID 76	Unknown	DQ491498	DQ470942	DQ471122
*M*. *fusca* (Pers.) P. Karst.	TNS:F17463/NBRC 112537	Japan	LC425049		
*Pezicula carpinea* (Pers.) Tul. ex Fuckel	CBS 282.39/AFTOL-ID 938	Canada	KR859272	DQ470967	DQ842032
*Vibrissea flavovirens* (Pers.) Korf & J.R. Dixon	CBS 121003	Germany	MT026430		
*V*. *truncorum* (Alb. & Schwein.) Fr.	CBS 258.91/AFTOL-ID 1322	Canada	MT026377	FJ176874	FJ238438

Note: Type specimens are indicated with superscript “T” and newly generated sequence data are indicated in black bold.

## Data Availability

Names of the new species were formally registered in the database Fungal Names (http://www.fungalinfo.net/fungalname/fungalname.html (accessed on 26 August 2021)). Specimens were deposited in the Herbarium Mycologicum Academiae Sinicae (HMAS). The newly generated sequences were deposited in GenBank (https://www.ncbi.nlm.nih.gov/genbank (accessed on 28 August 2021)).
